# Synchrotron radiation X-ray imaging with large field of view and high resolution using micro-scanning method

**DOI:** 10.1107/S1600577522007652

**Published:** 2022-08-12

**Authors:** Rui Sun, Yanping Wang, Jie Zhang, Tijian Deng, Qiru Yi, Bei Yu, Mei Huang, Gang Li, Xiaoming Jiang

**Affiliations:** a Institute of High Energy Physics, Chinese Academy of Sciences, 19B Yuquan Road, Shijingshan District, Beijing 100049, People’s Republic of China; b University of Chinese Academy of Sciences, 19A Yuquan Road, Shijingshan District, Beijing 100049, People’s Republic of China; University of Tokyo, Japan

**Keywords:** synchrotron radiation X-ray imaging, micro-scanning, oversampled image, point spread function (PSF)

## Abstract

In synchrotron radiation X-ray imaging, the imaging field of view and spatial resolution are mutually restricted, which makes it impossible to have both a large field of view and high resolution when carrying out experiments. Constructing an oversampled image through the micro-scanning method and using the deconvolution algorithm to eliminate the point spread function introduced by pixel overlap can increase the resolution under a fixed imaging field of view, thereby improving the ratio of the field of view to the spatial resolution. In this paper, numerical simulation and synchrotron radiation experiments are carried out with a different number of micro-scanning steps. In numerical simulation experiments only affected by the image pixel size, as the number of micro-scanning steps increases, the ability of the oversampled image with deconvolution to improve the resolution is stronger. The achievable resolution of the oversampled image with deconvolution is basically the same as that of the sample image. In the synchrotron radiation experiments, the resolution of the oversampled image with deconvolution in the 2 × 2 mode is significantly improved. However, as the number of micro-scanning steps increases, the resolution improvement is limited, or even no longer improved. Finally, by analyzing the results of numerical simulation and synchrotron radiation experiments, three factors (four other factors affecting the resolution besides the camera resolution, translational accuracy of micro-scanning, and the signal-to-noise ratio of projections) affecting the micro-scanning method are proposed and verified by experiments.

## Abstract 2

In synchrotron radiation X-ray imaging, the spatial resolution under a fixed field of view is improved using the micro-scanning method.

## 1. Introduction

X-ray imaging is one of the three mainstream technologies (diffraction, spectroscopy and imaging) of synchrotron radiation (Helliwell, 1998 ▸). In synchrotron radiation X-ray (SRX) imaging, many important studies require both a large field of view and high spatial resolution. For example, in whole brain mesoscopic imaging, a centimetre-level imaging field of view and a micrometre-level imaging spatial resolution are required. This means that the ratio of the visual field of view to the resolution must be more than 10000. At present, the number of pixels of an SRX imaging detector is mostly only 2k × 2k, and a resolution of 1 µm requires the effective pixel size of the detector to be less than 0.5 µm. At this time, the imaging field of view is only 1 mm × 1 mm, which cannot achieve centimetre-level field of view and micrometre-level resolution.

**Figure 1:**
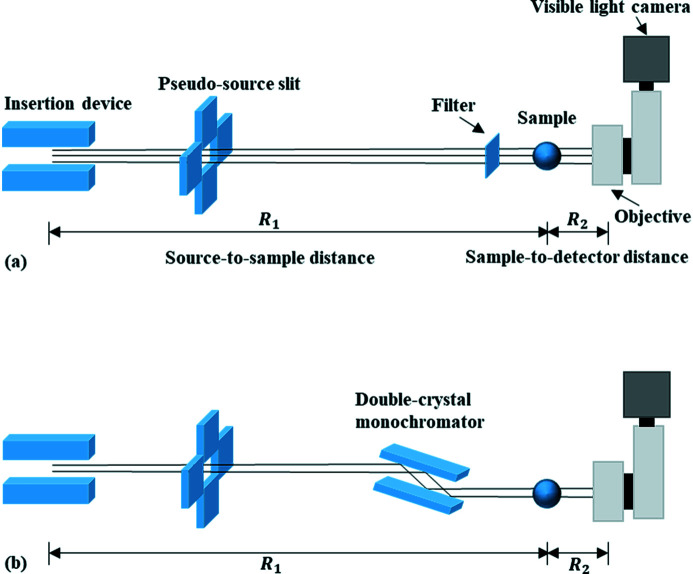
Schematic of the X-ray imaging experiment with synchrotron radiation (*a*) pink beam and (*b*) monochromatic beam.

**Figure 2:**
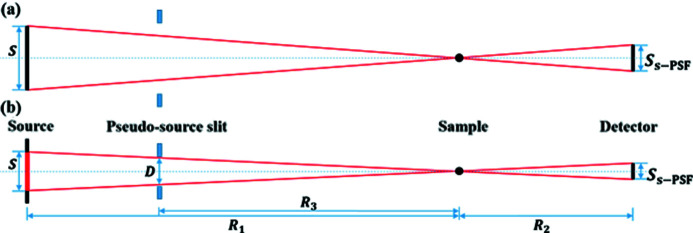
Principle diagram for calculating the size of the source PSF. (*a*) When the white-beam slit is fully opened, the intensity of any point on the sample comes from the contribution of each point on the entire source (black). In this case, the entire source size is the effective size. (*b*) When the slit size becomes smaller, the intensity comes from the contribution of each point on the effective source (red).

The lens-coupled X-ray indirect imaging detector can achieve resolutions from sub-micrometre to tens of micrometres. Currently, almost all SRX imaging experimental stations are equipped with this type of detection device (Gruner, 2012 ▸). This type of detector consists of a scintillator, a lens coupling system and a visible-light camera (Wang *et al.*, 2018 ▸). In Fig. 1 ▸(*a*), the pink beam with different main energy can be obtained by using different filters in the pink-beam imaging experiments. In Fig. 1 ▸(*b*), the double-crystal monochromator is used to monochromatize the white beam in monochromatic beam imaging experiments. Fresnel diffraction, the point spread function (PSF) of the source to the sample (abbreviated as the source PSF), the diffraction limit of the lens-coupled system, the scintillator, and the effective pixel size of the detector all affect the spatial resolution of imaging. The limitation of Fresnel diffraction on imaging resolution is expressed by the width of the first Fresnel zone as

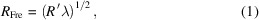

where λ is the wavelength of the incident X-rays, and *R*′ = *R*_2_/*M* is the effective propagation distance (Gureyev *et al.*, 2008 ▸; Wilkins *et al.*, 2014 ▸). *M* = (*R*_1_ + *R*_2_)/*R*_1_ is the geometric magnification, where *R*_1_ is the source-to-sample distance and *R*_2_ is the sample-to-detector distance. According to Fig. 2 ▸, the calculation formula of the size of the source PSF is

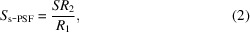

where *S* is the effective size of the source (Gureyev *et al.*, 2009 ▸). In particular, for the case of Fig. 2 ▸(*b*), the size of the source PSF can also be calculated by

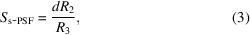

where *d* is the slit size and *R*_3_ is the slit-to-sample distance. If the source PSF is symmetrically distributed, the distance between two points whose intensity is 36.75% of the peak intensity on both sides of the source PSF peak represents the highest resolution *R*_src_ under this source PSF (Otón *et al.*, 2016 ▸). The formula for calculating the diffraction limit of the lens-coupled system is

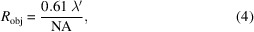

where λ′ is the wavelength of visible light after the X-rays are converted by the scintillator, and NA is the numerical aperture of the objective (Rayleigh, 1874 ▸). The expression of the detector depth of field is

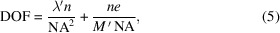

where *n* is the refractive index of air, *e* is the pixel size of the detector, and *M*′ is the magnification of the detector (Jones *et al.*, 2008 ▸). During the experiment, the thickness of the scintillator or the attenuation length of the scintillator to the incident X-rays should be smaller than the detector depth of field, otherwise the scintillator will reduce the spatial resolution of the imaging system. According to the Nyquist–Shannon sampling theorem, two times the effective pixel size *e*/*M*′ of the detector should be taken as the camera resolution, 

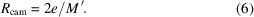

On the other hand, the beam size at the sample position, the magnification and the field in the image space of the lens-coupled system, as well as the pixel number and size of the visible-light camera, affect the imaging field of view. The beam size at the sample position is determined by the divergence angle and the source-to-sample distance. Under the premise of the fixed number of detector pixels, a large field of view and high resolution are contradictory indicators, which makes them impossible to achieve at the same time. To improve the spatial resolution of an image, a coupling system with a larger magnification or a camera with a smaller pixel size must be used, which will reduce the imaging field of view.

The methods to solve the above contradiction include developing large-field-of-view detectors (Komatsu *et al.*, 1993 ▸) and adopting the micro-scanning method. The development of a large-field-of-view detector is difficult and expensive. The micro-scanning method can realize SRX imaging with large field of view and high resolution at the same time without increasing the number of detector pixels. Micro-scanning refers to scanning a two-dimensional image with sub-pixel distance as the step size and fusing multiple sequential images of the same sample to construct an oversampling image. According to whether the micro-scanning step size is fixed or not, it can be divided into controlled micro-scanning and uncontrolled micro-scanning. In 1986, Dann *et al.* (1986 ▸) first proposed the concept of ‘micro-scanning’. Blommel *et al.* (1991 ▸) introduced the micro-scanning method in detail. Micro-scanning is mainly used in imaging to improve the image resolution or system modulation transfer function (MTF) while keeping the field of view unchanged. Bruandet & Dinten (1999 ▸) applied this method to the X-ray imaging field and improved the MTF of the imaging system (pixel size: 200 µm × 200 µm). Thim *et al.* (2011 ▸) increased the resolution to 1.8 times when using a single-photon processing system with pixel size of 220 µm × 220 µm and micro-scanning step size of 1/4 pixel size. They increased the resolution to 1.67 times when using a single-photon processing system with pixel size of 55 µm × 55 µm and micro-scanning step size of 1/3 pixel size. Ehn *et al.* studied the influence of the micro-scanning step size on the MTF and applied it to X-ray computed tomography (CT) imaging (Ehn *et al.*, 2016 ▸; Ballabriga *et al.*, 2013 ▸; Pennicard *et al.*, 2013 ▸). Lübcke *et al.* (2019 ▸) used a synchrotron radiation soft X-ray detector (effective pixel size: 7.2 µm × 7.2 µm) to increase the image spatial resolution to 1.3 times by uncontrolled micro-scanning with a CCD chip. Lifton & Liu (2020 ▸) studied the application of the micro-scanning method under the cone beam in X-ray CT imaging. Using the SRX source and the lens-coupled indirect imaging detector, this article adopts controlled micro-scanning to improve the spatial resolution under a fixed imaging field of view, to increase the ratio of the field of view to the spatial resolution. After analyzing the experimental results, the factors affecting the resolution improvement effect are given.

## 2. Principle of oversampling imaging based on controlled micro-scanning

Micro-scanning can be seen as an oversampling process. First, a sequence of projections of the sample is obtained by controlled micro-scanning with horizontal and vertical number of micro-scanning steps of *m* and *n* (*m* and *n* are both positive integers), respectively. So the horizontal and vertical micro-scanning step size is 1/*m* and 1/*n* of the pixel size of the projection (or the effective pixel size of the detector), respectively. Then, these projections are merged according to the scan position to construct an oversampled image. The strong correlation between adjacent pixels in the oversampled image caused by the overlap of pixels is related to the number of micro-scanning steps and the effective pixel size of the detector. This strong correlation can be called the PSF with oversampled image (PSF_px_). Ehn *et al.* explain in detail how to determine PSF_px_ (Ehn *et al.*, 2016 ▸). The matrix expression of this PSF_px_ is[Chem scheme1] 
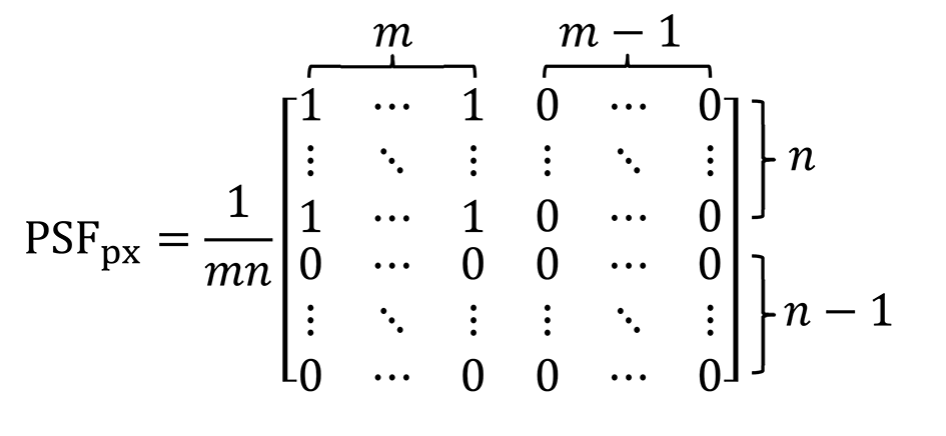


**Figure 3:**
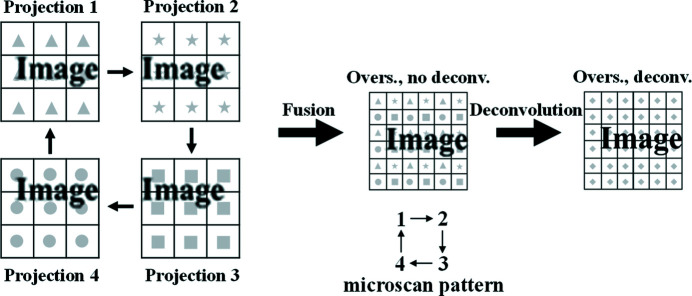
Representation of the 2 × 2 micro-scanning process.

The size of the PSF_px_ matrix elements is equal to the micro-scanning step size and the number of PSF_px_ matrix elements is (2*m* − 1) × (2*n* − 1). After the PSF_px_ is determined, one can apply a deconvolution method, such as the direct demodulation method (Li & Wu, 1994 ▸), to restore the latent image. The direct demodulation method uses an iterative algorithm to perform deconvolution operations under certain physical constraints. The iterative formula used in this paper is the Lucy–Richardson iterative formula (Richardson, 1972 ▸; Lucy, 1974 ▸). In this paper, deconvolution is used only to remove PSF_px_ to show the effect of the micro-scanning method. The 2 × 2 mode means that the micro-scanning step size is 1/2 pixel size of projection in the horizontal and vertical. The implementation process is shown in Fig. 3 ▸ (Fortin *et al.*, 1994 ▸).

In summary, the *m* × *n* mode micro-scanning imaging method includes the following steps:

(*a*) Using the micro-scanning method to obtain *m* × *n* sequence projections.

(*b*) Fusing the projections according to the micro-scanning position to obtain an oversampled image.

(*c*) Using the direct demodulation method to restore the latent image.

## 3. Experiments

### 3.1. Numerical simulation experiments

**Figure 4:**
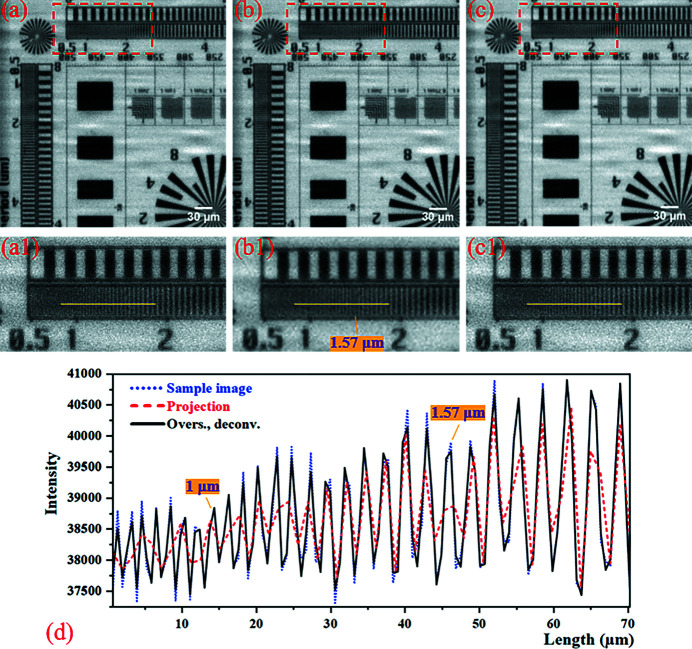
Results of the 2 × 2 mode numerical simulation experiment. (*a*) The sample image (pixel size: 0.65 µm × 0.65 µm; pixel number: 588 × 588). (*b*) The projection (pixel size: 1.3 µm × 1.3 µm; pixel number: 294 × 294). (*c*) The oversampled image with deconvolution (pixel size: 0.65 µm × 0.65 µm; pixel number: 588 × 588). Panels (*a*)–(*c*) are only part of the image to show the details more clearly. Panels (*a*1)–(*c*1) correspond to the magnified images in the red boxes of (*a*)–(*c*), respectively. (*d*) The intensity profiles at the yellow line of (*a*1)–(*c*1). It is important to note that the numbers marked on the image represent the line-width. The numerical annotations on all subsequent figures refer to the line-width.

The simulation experiment steps of the 2 × 2 mode micro-scanning imaging method are as follows. A high-resolution image (pixel size: 0.65 µm × 0.65 µm; pixel number: 2048 × 2048) was taken as the sample. This image was obtained by photographing the Xradia test pattern using 12 keV X-rays selected by a Si(111) double-crystal monochromator and a lens-coupled X-ray imaging detector with 10× objective (NA: 0.4) and Hamamatsu Flash 4.0 camera (pixel size: 6.5 µm × 6.5 µm; pixel number: 2048 × 2048) at the X-ray Imaging and Biomedical Applications beamline (BL13W1) of Shanghai Synchrotron Radiation Facility (SSRF), as shown in Fig. 4 ▸(*a*). Table 1 ▸ lists the design parameters of the SSRF BL13W1 beamline. The Xradia test pattern (X500-200-16, Xradia Inc., now Zeiss) has a line-width between 0.5 µm and 8 µm, *i.e.* a line-pitch between 1 µm and 16 µm (Douissard *et al.*, 2012 ▸; Maier *et al.*, 2017 ▸). The relevant parameters of this Xradia test pattern are shown in Table 2 ▸. Firstly, the average intensity of the four pixels in the 2 × 2 window is taken as the intensity of the corresponding pixel of the projection image (pixel size: 1.3 µm × 1.3 µm; pixel number: 1024 × 1024). Secondly, the 1/2 pixel size of the projection is taken as the step size of the controlled micro-scanning, and the micro-scanning sequence is as shown in Fig. 3 ▸. Four simulated projections that are offset by half a pixel with each other are obtained. Then, the four projections are fused to obtain an oversampled image. Finally, the PSF_px_ of the oversampled image is eliminated by the direct demodulation method to obtain the final high-pixel-number and high-resolution image (pixel size: 0.65 µm × 0.65 µm; pixel number: 2048 × 2048). By analogy, the simulation experiment steps of 3 × 3, 4 × 4 and other modes can be given.

The results of the 2 × 2 mode numerical simulation experiment are shown in Fig. 4 ▸. Comparing Figs. 4 ▸(*b*) and 4 ▸(*c*), it can be seen that using the micro-scanning method the image resolution and contrast of the oversampled image with deconvolution are improved without reducing the imaging field of view. Then, comparing Figs. 4 ▸(*a*) and 4 ▸(*c*), the resolution of the oversampled image with deconvolution is restored to be basically the same as that of the sample image. As shown in Fig. 4 ▸(*d*), the contrast of the projection deteriorated at 1.57 µm line-width, and the grating structure of the sample is aliased in the 1–30 µm region. However, the oversampled image with deconvolution improves the contrast and resolves the grating structure in the 1–30 µm region. At the same time, the profile of the oversampled image with deconvolution and the sample image in Fig. 4 ▸(*d*) basically coincide, reflecting that the resolution level of the oversampled image with deconvolution is restored to the same level as the sample image.

**Figure 5:**
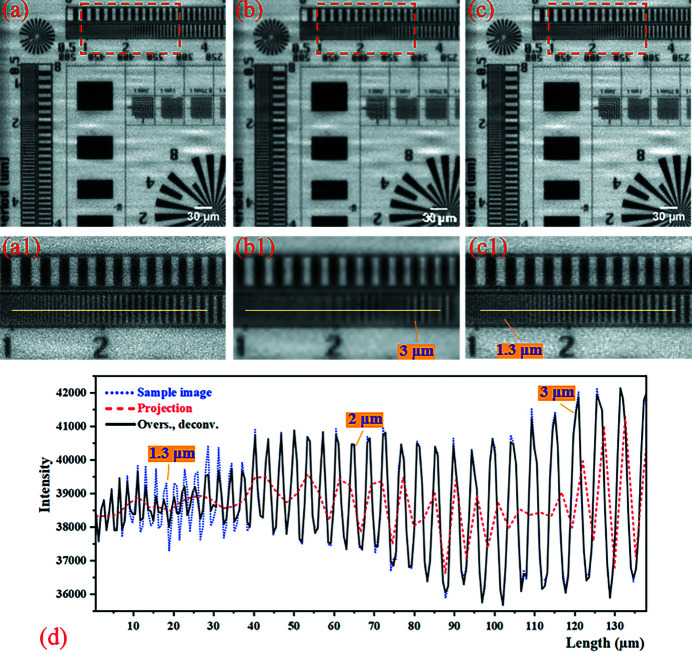
Results of the 4 × 4 mode numerical simulation experiment. (*a*) The sample image (pixel size: 0.65 µm × 0.65 µm; pixel number: 588 × 588). (*b*) The projection (pixel size: 2.6 µm × 2.6 µm; pixel number: 147 × 147). (*c*) The oversampled image with deconvolution (pixel size: 0.65 µm × 0.65 µm; pixel number: 588 × 588). Panels (*a*)–(*c*) are only part of the image to show the details more clearly. Panels (*a*1)–(*c*1) correspond to the magnified images in the red boxes of (*a*)–(c), respectively. (*d*) The intensity profiles at the yellow line of (*a*1)–(*c*1).

**Figure 6:**
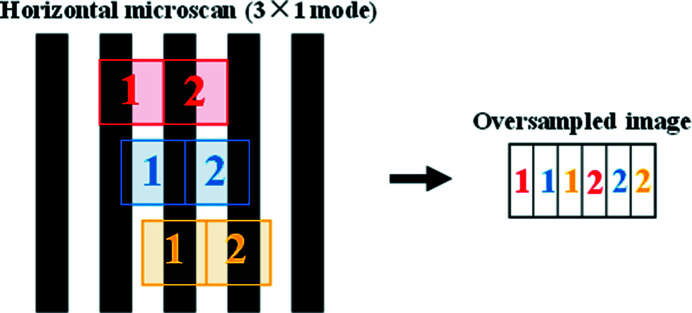
A special case where the micro-scanning method fails. The vertical position of the micro-scan was intentionally staggered for clear observation.

Following the same method, the 4 × 4 mode micro-scanning method was numerically simulated, and the results are shown in Fig. 5 ▸. Comparing Figs. 5 ▸(*b*) and 5 ▸(*c*), it can be seen that the image resolution and contrast of the oversampled image with deconvolution are greatly improved by using the micro-scanning method without reducing the imaging field of view. The ability to improve the resolution of the 4 × 4 mode micro-scanning method is much greater than that of the 2 × 2 mode. However, comparing Figs. 5 ▸(*a*) and 5 ▸(*c*), it is found that the oversampled image with deconvolution is visually blurred at about 1.3 µm line-width. The experimental results were further analyzed in Fig. 5 ▸(*d*). The sample structure in the projection begins to alias from the 3 µm line-width. In addition to the poor recovery at about 1.3 µm line-width, the 4 × 4 mode oversampling image with deconvolution has greatly improved resolution and contrast, and basically reaches the resolution level of the sample image. As shown in Fig. 5 ▸(*d*), the degree of contrast recovery of the oversampled image with deconvolution in the 10–30 µm region is even worse than that at the higher resolution (1–10 µm region), which is related to the grating structure of the sample. As shown in Fig. 6 ▸, when the effective pixel size of the detector is almost the same as the line-pitch of the grating, the strong correlation (intensity difference) between adjacent pixels of the oversampled image cannot be obtained by the micro-scanning method, which leads to failure of this method. In the 4 × 4 mode numerical simulation experiment, the pixel size of the projection is 2.6 µm, and the strong correlation caused by the micro-scanning method at 2.6 µm line-pitch (1.3 µm line-width) is very weak. So the oversampled image with deconvolution has poor contrast recovery around here.

In conclusion, numerical simulation experiments that exclude all error factors demonstrate that the micro-scanning method improves the spatial resolution while keeping the imaging field of view unchanged, thereby increasing the ratio of the field of view to spatial resolution. In the numerical simulation experiments only affected by the image pixel size, the higher the number of micro-scanning steps, the greater the degree of resolution improvement.

### 3.2. SRX imaging experiments

#### 3.2.1. The Xradia test pattern

**Figure 7:**
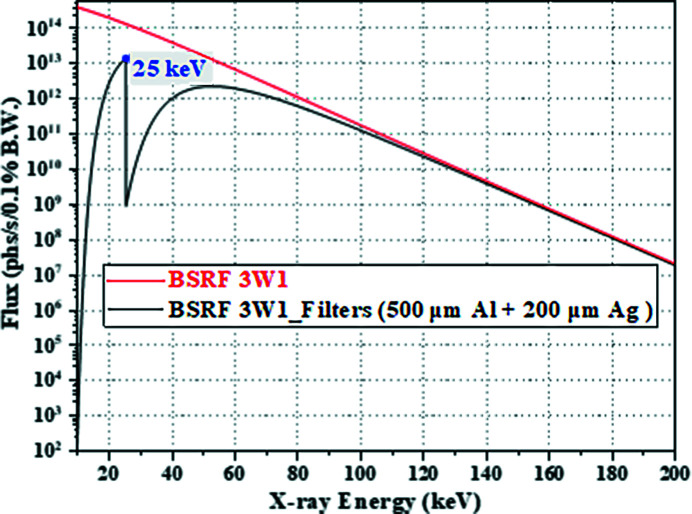
Spectrum of the pink beam converted from the white beam of the BSRF 3W1 beamline using two filters (500 µm-thick Al and 200 µm-thick Ag) together.

The experiments were carried out at the 3W1 beamline station of the Beijing Synchrotron Radiation Facility (BSRF). The design parameters of the BSRF 3W1 beamline are shown in Table 1 ▸. The white beam is from the superconducting wiggler, and two filters, 500 µm-thick Al and 200 µm-thick Ag, were used to turn it into pink beam together. The spectrum of this pink beam with main energy of 25 keV is shown in Fig. 7 ▸. The full width at half-maximum (FWHM) of the source size is 3130 µm (horizontal) × 104 µm (vertical) at the superconducting wiggler. The slit size in the horizontal is set to 200 µm and the slit in the vertical is fully opened. This means that the effective source size in the horizontal direction is reduced [Fig. 2 ▸(*b*)], while the vertical direction [Fig. 2 ▸(*a*)] is not affected. The source-to-slit distance is 17.5 m. The experimental sample is still the Xradia test pattern, which was installed on the piezoelectric ceramic two-dimensional nano-translation stage (production company: Sanying Motion Control Technology Ltd; model: NS-XY100-01). The source-to-sample distance is 28 m and the sample-to-detector distance is 0.035 m. The lens-coupled detector consists of the scintillator with 50 µm-thick YAG:Ce, 5× objective (NA: 0.14) and the camera (iXon Ultra 888, Andor) with pixel number of 1024 × 1024 and pixel size of 13 µm × 13 µm. The YAG:Ce scintillator can convert X-rays into visible light with a peak wavelength of 550 nm (Tous *et al.*, 2013 ▸). However, the magnification is not accurate due to the short working distance of the visible-light conversion unit, so the effective pixel size of the detector is not theoretically 2.6 µm but 2.94 µm. The experiments used micro-scanning modes of 2 × 2, 3 × 3 and 4 × 4. During the experiment, each micro-scanning step was used to obtain a projection with a constant exposure time. Before and after each experiment, images without sample with the same exposure time were taken to eliminate the influence of beam unevenness during data processing.

**Figure 8:**
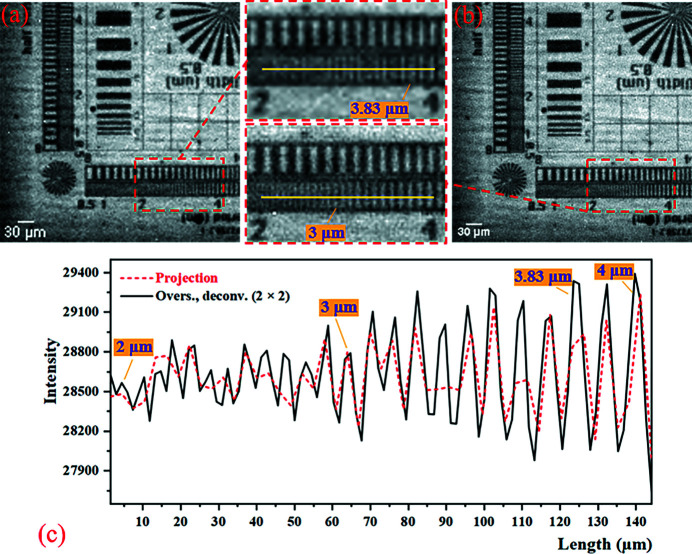
Results of the 2 × 2 mode SRX experiment. (*a*) The projection (pixel size: 2.94 µm × 2.94 µm; pixel number: 148 × 148). (*b*) The oversampled image with deconvolution (pixel size: 1.47 µm × 1.47 µm; pixel number: 296 × 296). Panels (*a*) and (*b*) are only part of the image to show the details more clearly. (*c*) The intensity profiles at the yellow line (line-width between 2 µm and 4 µm).

**Figure 9:**
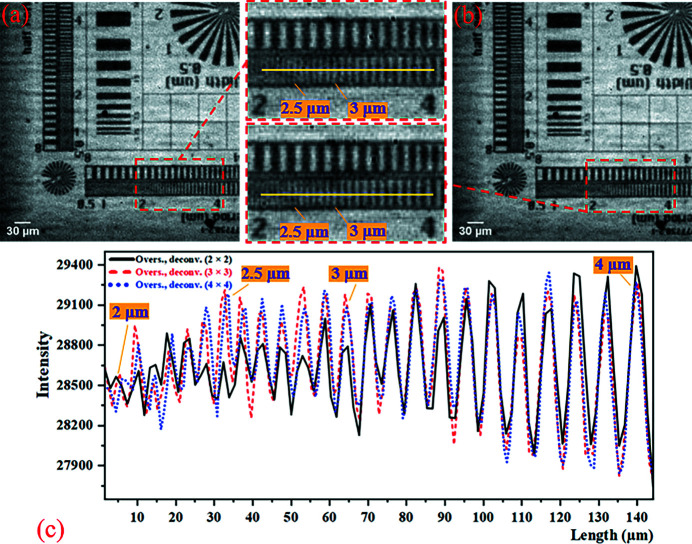
Results of the 3 × 3 and the 4 × 4 mode SRX experiments. (*a*) The 3 × 3 mode oversampled image with deconvolution (pixel size: 0.98 µm × 0.98 µm; pixel number: 444 × 444). (*b*) The 4 × 4 mode oversampled image with deconvolution (pixel size: 0.735 µm × 0.735 µm; pixel number: 592 × 592). Similarly, panels (*a*) and (*b*) are only part of the image to show the details more clearly. (*c*) The intensity profiles at the yellow line (the line-width between 2 µm and 4 µm) in 2 × 2, 3 × 3 and 4 × 4 micro-scanning mode.

According to formulas (1)–(6), the calculation results of the five factors affecting the imaging resolution of this experiment are shown in Table 3 ▸. The value of the camera resolution determined by the effective pixel size is much larger than the others, so the resolution of the projection is determined by the effective pixel size of the detector. The thickness of the scintillator is very close to the depth of field (DOF), so the scintillator does not affect the imaging resolution. The results of the 2 × 2 mode SRX imaging experiment are shown in Fig. 8 ▸. Comparing Figs. 8 ▸(*a*) and 8 ▸(*b*), it can be seen that the 2 × 2 mode micro-scanning method applied to synchrotron radiation imaging has a very obvious effect of improving the resolution without reducing the imaging field of view. As shown in Fig. 8 ▸(*c*), the contrast of the projection becomes poor at the 3.83 µm line-width, and the sample structure begins to alias. The resolution of the oversampled image with deconvolution in the 2 × 2 mode can reach at least 3 µm line-width, and the contrast of the resolution that can be achieved in the projection is improved. The results of the 3 × 3 and the 4 × 4 mode SRX imaging experiments are shown in Fig. 9 ▸. Between 2.5 µm and 3 µm line-width, the contrast of the 3 × 3 mode image is slightly improved compared with the 2 × 2 mode image. But the 4 × 4 mode image shows no improvement over the 3 × 3 mode. In conclusion, the synchrotron radiation experiments using the micro-scanning method can significantly improve the image resolution and contrast of the 2 × 2 mode without reducing the imaging field-of-view. But as the number of micro-scanning steps increased, the resolution of the image did not improve much.

#### 3.2.2. Animal sponge

**Figure 10:**
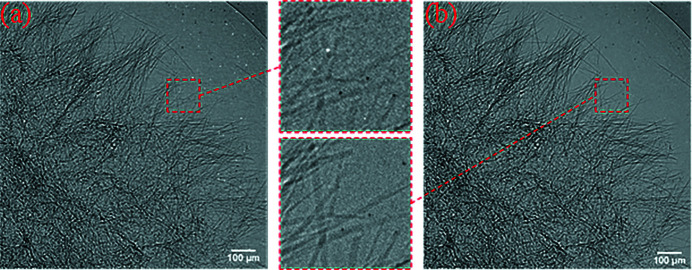
Results of the 2 × 2 mode SRX experiment with the animal sponge. (*a*) The projection (pixel size: 1.43 µm × 1.43 µm; pixel number: 840 × 840). (*b*) The oversampled image with deconvolution (pixel size: 0.715 µm × 0.715 µm; pixel number: 1680 × 1680). Panels (*a*) and (*b*) are only part of the image to show the details more clearly.

In order to verify whether the micro-scanning method is effective for general applications, dehydrated animal sponge was used as a test sample. The experiment was also carried out at the BSRF 3W1 beamline, and the source and the experimental devices were the same as that of the Xradia pattern experiments except for the visible-light camera. The visible-light camera used in this experiment is a Hamamatsu Flash 4.0. Since there is also the problem of working distance, the effective pixel size of the detector is 1.43 µm × 1.43 µm. The experiment adopts the 2 × 2 mode micro-scanning method, and the experimental results are shown in Fig. 10 ▸. The 2 × 2 mode oversampled image with deconvolution has significantly better contrast than the projection. It can be seen from the magnified image in the red box that the sample structure that is not easily distinguishable in the projection can be clearly observed after 2 × 2 mode micro-scanning.

## 4. Analysis and discussion

The essential principle of the micro-scanning method to construct an oversampling image is to reduce the effective pixel size of the detector, so as to obtain more high-frequency information to improve the spatial resolution of the image. Several factors affecting the micro-scanning method in synchrotron radiation experiments are proposed.

**Figure 11:**
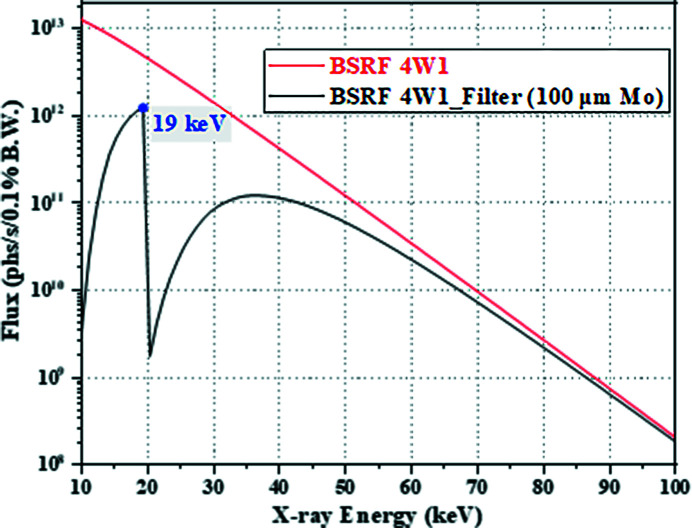
The spectrum of the pink beam was converted from the white beam of the BSRF 4W1A beamline using a 100 µm-thick Mo filter.

(1) *Four other factors affecting the resolution besides the pixel size.* When the effect of any of the four factors on the resolution cannot be ignored relative to the pixel size of the oversampled image, the ability of improving the resolution by the micro-scanning method will be worse, and sometimes even not reflected at all. In this regard, the 2 × 2 micro-scanning experiment of the Xradia test pattern was carried out at the BRSF 4W1A beamline station for verification. The design parameters of the BSRF 4W1A beamline are shown in Table 1 ▸. The white beam is from the wiggler, and the filter of 100 µm-thick Mo was used to turn it into pink beam. The spectrum of this pink beam with main energy of 19 keV is shown in Fig. 11 ▸. The FWHM of the source size is 1230 µm (horizontal) × 420 µm (vertical) at the superconducting wiggler. The slit is fully opened in the horizontal and vertical. The source-to-sample distance is 42 m and the sample-to-detector distance is 0.1 m. The lens-coupled detector consists of the scintillator with 50 µm-thick YAG:Ce, 2× objective (NA: 0.055) and camera (Hamamatsu Flash 4.0). Since the visible-light conversion units with the short working distance used in the previous experiments is still used, the effective pixel size of the detector is 3.57 µm × 3.57 µm.

**Figure 12:**
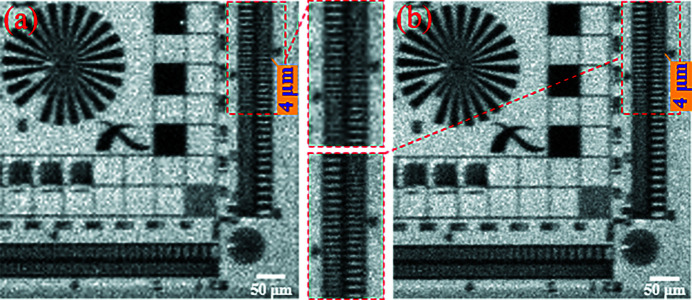
Experimental results verifying the effect of the source PSF in the horizontal on the micro-scanning method. (*a*) The projection (pixel size: 3.57 µm × 3.57 µm; pixel number: 134 × 134). (*b*) The oversampled image with deconvolution (pixel size: 1.785 µm × 1.785 µm; pixel number: 268 × 268). Panels (*a*) and (*b*) are only part of the image to show the details more clearly.

According to formulas (1)–(6), the calculation results of the five factors affecting the imaging resolution of this experiment are shown in Table 4 ▸. The experimental results are shown in Fig. 12 ▸. Comparing Figs. 12 ▸(*a*) and 12 ▸(*b*), vertically, the projection is blurred at the 4 µm line-width, while structure can be observed below the 4 µm line-width in the oversampled image with deconvolution. This proves that under these experimental conditions the micro-scanning method can improve the spatial resolution in the vertical. However, the horizontal resolution of the oversampled image with deconvolution does not improve in any way. For projections, the combined limit of Fresnel diffraction, the source PSF and the diffraction limit on resolution is smaller in the vertical direction than the limit of the effective pixel size on resolution, and the opposite is true in the horizontal direction. This means the vertical resolution depends on the effective pixel size, while the horizontal resolution does not. Therefore, the vertical resolution is increased when using the micro-scanning method, while the resolution in the horizontal is basically unchanged. When using the micro-scanning method, it is necessary to avoid factors other than the camera resolution that affect the imaging resolution.

(2) *Translational accuracy of micro-scanning.* In the process of micro-scanning, the different translational accuracy will result in different shapes of PSF_px_. A larger translational error of the micro-scanning will cause a larger difference between the real PSF_px_ and the theoretical PSF_px_, thus affecting the result of deconvolution. For imaging experiments under visible light, the image signal-to-noise ratio (SNR) is better, and only two factors, diffraction limit and image pixel size, affect the imaging resolution. Therefore, the micro-scanning experiments were carried out under visible light to verify the influence of the translational error. A motion estimation algorithm based on scale invariant feature transformation (SIFT) and random sample consensus (RANSAC) is used to calculate the deviation of the actual translational distance of the projections from the ideal translational distance (Wang *et al.*, 2008 ▸). For example, in the 2 × 2 mode micro-scanning experiment, referring to the scanning sequence in Fig. 3 ▸, the projection 2 only moves horizontally and does not move vertically relative to the projection 1. Therefore, the ideal translational distance of projection 2 relative to projection 1 is 0.5 pixel size in the horizontal and 0 pixel size in the vertical.

**Figure 13:**
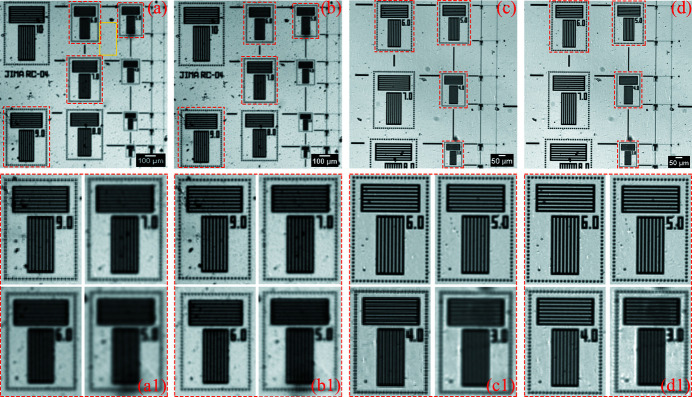
Experimental results verifying the effect of the translational accuracy on the micro-scanning method. (*a*) The projection (pixel size: 6.5 µm × 6.5 µm; pixel number: 230 × 230) and (*b*) the oversampled image with deconvolution (pixel size: 3.25 µm × 3.25 µm; pixel number: 460 × 460) in the case of high translational accuracy. (*c*) The projection (pixel size: 3.25 µm × 3.25 µm; pixel number: 360 × 360) and (*d*) the oversampled image with deconvolution (pixel size: 1.625 µm × 1.625 µm; pixel number: 720 × 720) in the case of poor translational accuracy. Panels (*a*)–(*d*) are only part of the image to show the details more clearly. Panels (*a*1)–(*d*1) correspond to the magnified images in the red boxes of panels (*a*)–(*d*), respectively.

The detector of the visible-light experiments uses the lens-coupled detector without the scintillator. The visible-light camera in the lens-coupled detector is an iXon Ultra 888. The sample is an RT RC-04 test pattern manufactured by JIMA, with line-width between 0.1 µm and 10 µm and line-pitch between 0.2 µm and 20 µm. Micro-scanning imaging experiments in 2 × 2 mode were carried out using 2× and 4× objectives. The first two rows of Table 5 ▸ list the experimental conditions and the translational errors of the two groups of experiments. The micro-scanning step size is the pixel size of the oversampled image. In these two sets of experiments, the determined camera resolutions of the oversampled images are both lower than the diffraction limit, so the influence of the diffraction limit on the micro-scanning method can be ruled out. In order to make the SNR of the projections taken with the 2× and 4× objectives close, the appropriate exposure time is chosen. In both sets of experiments, the experimental results are shown in Fig. 13 ▸. For the 2× objective, the micro-scanning step size is 3.25 µm, and the maximum translational error is 0.01 pixel size in the horizontal and 0.02 pixel size in the vertical, accounting for 2% and 4% of the pixel size of the oversampled image, respectively. In Fig. 13 ▸(*a*), the projection can clearly observe the 10 µm line-width, and the grating structure no longer has the same width at 9 µm line-width in the projection. In Fig. 13 ▸(*b*), the oversampled image with deconvolution can roughly observe the 5 µm line-width. Therefore, when the micro-scanning step size is 3.25 µm (2× objective), the image resolution is increased to nearly two times the original projection. Under the conditions of the 4× objective, the micro-scanning step size is 1.625 µm, and the maximum translational error is 0.05 pixel size in the horizontal and 0.07 pixel size in the vertical, accounting for 10% and 14% of the pixel size of the oversampled image, respectively. The resolution is improved from 5 µm line-width [Fig. 13 ▸(*c*)] to 3 µm line-width [Fig. 13 ▸(*d*)], which is about 1.67 times the original projection. When the micro-scanning step size is smaller, translational errors will increase, which will make the effect of resolution improvement worse by using the micro-scanning method with a fixed field of view.

**Figure 14:**
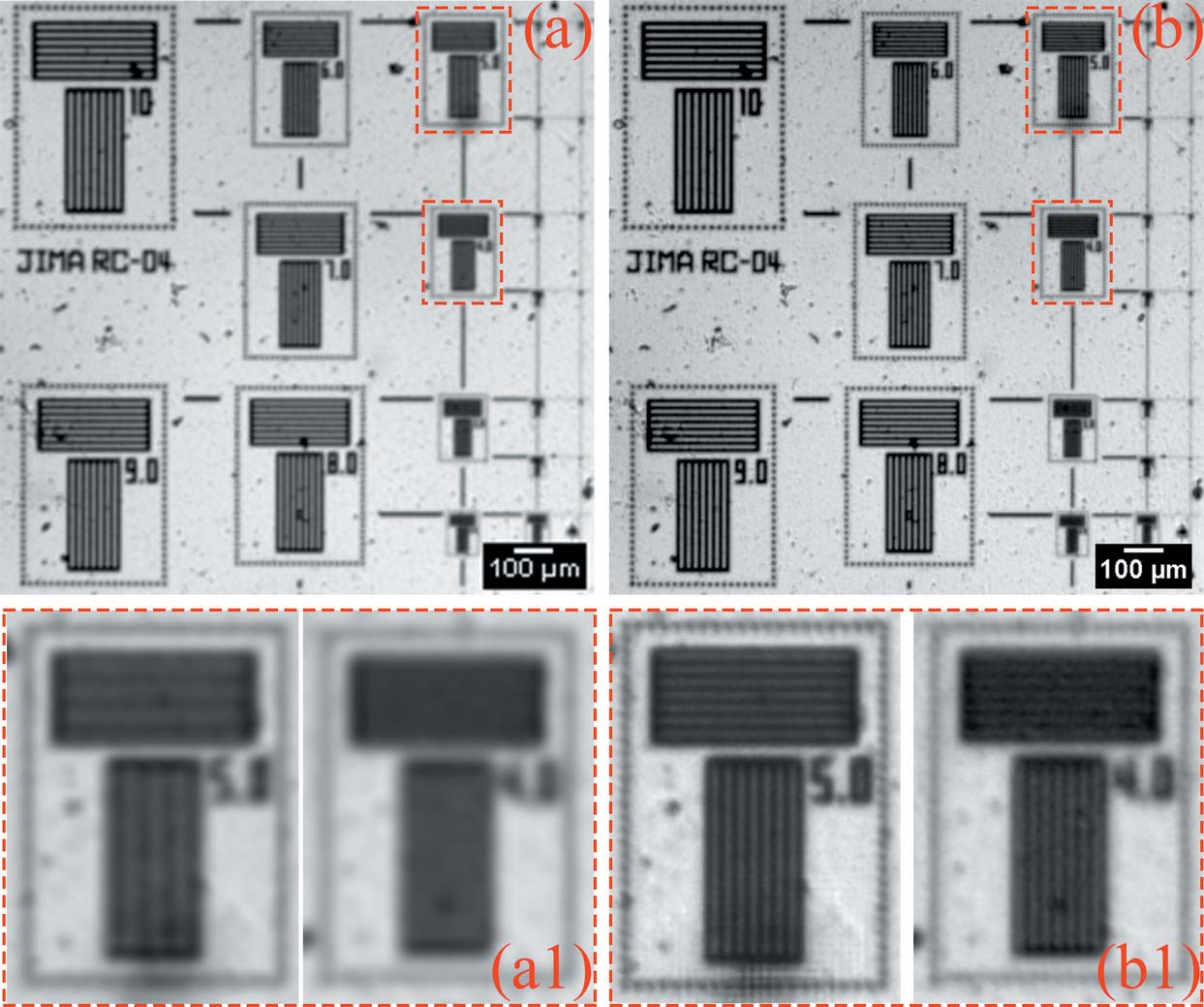
The 3 × 3 mode experimental results of using visible light. (*a*) The projection (pixel size: 6.5 µm × 6.5 µm; pixel number: 230 × 230). (*b*) The oversampled image with deconvolution (pixel size: 2.17 µm × 2.17 µm; pixel number: 690 × 690). Panels (*a*) and (*b*) are only part of the image to show the details more clearly. Panels (*a*1) and (*b*1) correspond to the magnified images in the red boxes of (*a*) and (*b*), respectively.

At the same time, the 3 × 3 mode micro-scanning experiment with visible light was also carried out using the 2× objective. The experimental conditions and the translational errors are listed in the third row of Table 5 ▸. In this set of experiments, the camera resolution determined by the pixel size of the oversampled image is lower than the diffraction limit, so the influence of the diffraction limit on the micro-scanning method can also be ruled out. Since only the scanning mode is changed in this set of comparative experiments, the influence of the image SNR does not need to be considered. The micro-scanning step size of this experiment is 2.17 µm. The experimental results are shown in Fig. 14 ▸. In the process of 3 × 3 mode micro-scanning, two translational operations are carried out in the horizontal and vertical directions, respectively, and the translational errors in each direction will be accumulated. This results in a maximum translational error of 0.04 pixel size in the horizontal and 0.08 pixel size in the vertical, accounting for 12% and 24% of the pixel size of the oversampled image, respectively. As shown in Fig. 14 ▸, it is a little difficult to distinguish the 4 µm line-width of the oversampled image with deconvolution in the 3 × 3 mode micro-scanning experiment. The higher the number of micro-scanning steps, the larger the translational error of the projection accumulated. This makes the resolution improvement of 3 × 3 mode relative to 2 × 2 mode not so obvious.

**Figure 15:**
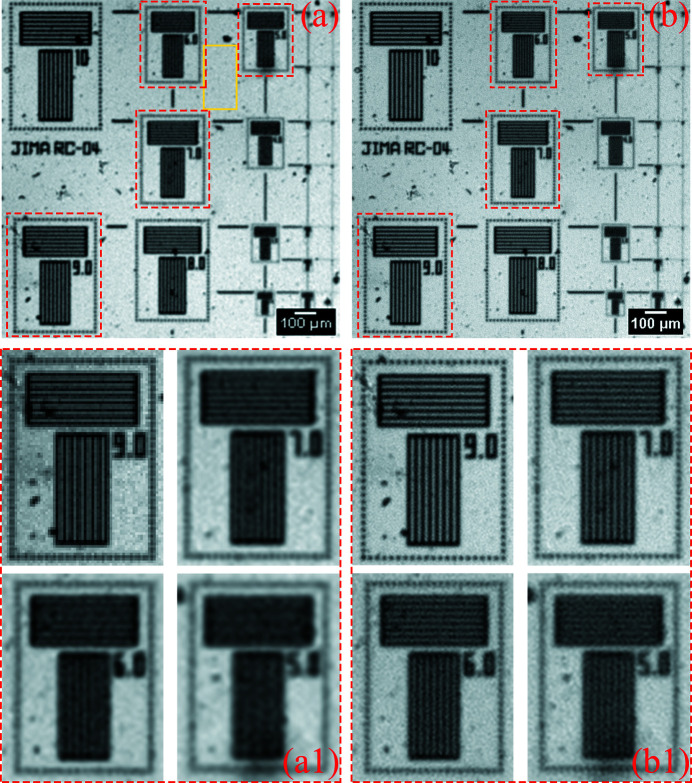
Experimental results verifying the effect of the projection SNR on the micro-scanning method. (*a*) The projection of low SNR (pixel size: 6.5 µm × 6.5 µm; pixel number: 230 × 230). (*b*) The oversampled image with deconvolution of low SNR (pixel size: 3.25 µm × 3.25 µm; pixel number: 460 × 460). Panels (*a*) and (*b*) are only part of the image to show the details more clearly.

(3) *SNR of projections.* For an image, the higher the required resolution of the sample structure, the lower its contrast. At this time, if the image noise or the intensity fluctuation between the projection images is too large, the strong correlation between the adjacent pixels of the oversampled image will be drowned out. The image SNR was reduced by adding noise to the four projections in the experiment shown in Fig. 13 ▸(*a*) to verify the influence of SNR on the micro-scanning method. In this way, the first two factors, namely four other factors affecting the resolution besides the pixel size and translational accuracy, will not affect this group of micro-scanning experiments. After adding noise, the standard deviation of the background noise in the yellow box changes from 82 in Fig. 13 ▸(*a*) to 105 in Fig. 15 ▸(*a*). Therefore, with the original image without added noise as the reference image, the SNR of the noise-added image becomes worse. Fig. 15 ▸ shows the results of the 2 × 2 mode micro-scanning experiment with low projection SNR. The oversampled image with deconvolution [Fig. 15 ▸(*b*)] can resolve up to 6 µm line-width, which is worse than the original oversampled image with deconvolution [Fig. 13 ▸(*b*)]. The ability to use the micro-scanning method to increase resolution at low projection SNR is greatly reduced.

Based on the analysis of the above three influencing factors, in the SRX imaging experiments of the Xradia test pattern of the previous section, the two factors of image SNR and micro-scanning translational accuracy will decrease the effect of micro-scanning to improve the resolution. In the experiment, the resolution of 3 × 3 mode is very slightly improved compared with that of 2 × 2 mode, and the resolution of 4 × 4 mode is not improved compared with that of 3 × 3 mode. The direct reason for this phenomenon is that the diffraction limit in the 3 × 3 mode and 4 × 4 mode is already lower than the camera resolution determined by the pixel size of the oversampled image, which limits the improvement of the resolution.

## 5. Conclusion

In this work, we have successfully demonstrated the potential of using the micro-scanning method to improve the ratio of the imaging field of view to the spatial resolution of the imaging system, which is of great significance for the development of large-field-of-view high-resolution SRX imaging experiments. First, numerical simulation experiments without any experimental errors were carried out to verify the micro-scanning method. The results show that the oversampled image with deconvolution obtained by the micro-scanning method can effectively improve the resolution without reducing the field of view, and the resolution level it can achieve is consistent with the sample image. At the same time, the ability of the 4 × 4 mode to increase the resolution is much higher than that of the 2 × 2 mode. Then, micro-scanning experiments were carried out using synchrotron radiation. Experimental results show that the oversampled image with deconvolution can improve the spatial resolution in the fixed field of view. In the 2 × 2 mode, the effect of resolution improvement is very obvious. The result of the 3 × 3 mode is slightly improved over the 2 × 2 mode, but the result of the 4 × 4 mode is basically no improvement over the 3 × 3 mode. Aiming at the phenomenon that the experimental results of synchrotron radiation are quite different from those of numerical simulation experiments, we propose three factors (four other factors affecting the resolution besides the camera resolution, translational accuracy of micro-scanning and the SNR of projections) that affect the micro-scanning method and verify them by experiments. In synchrotron radiation experiments using the Xradia test pattern, the resolution improvement of the oversampled image is limited by the diffraction limit, which leads to the resolution improvement reaching the limit with an increase of the number of micro-scanning steps.

When using the micro-scanning method to increase the resolution, we should first consider whether the effective pixel size of the detector limits the resolution of the projection. In addition, the translational accuracy and repeatability of the micro-scanning must be sufficiently high. The PSF_px_ used to eliminate the strong correlation between adjacent pixels of an oversampled image relies on the equidistance of the sequence projections and the repeatability of the micro-scanning. The measurement time and radiation dose during the experiment are accumulated according to the number of projections, but the resolution is not blindly improved. Therefore, the optimal number of micro-scanning steps should be selected for imaging experiments under the conditions of small error of the micro-scanning step size and great image SNR. Among them, the influence of quantization translational error and image SNR on the micro-scanning method is important and urgent work. The next step will be to supplement and improve this part of the work.

## Figures and Tables

**Table 1:** Design parameters of the SSRF BL13W1 beamline, the BSRF 3W1 beamline and 4W1A beamline; the source size is the full width at half-maximum (FWHM) of the electron beam at the wiggler

Parameter	SSRF BL13W1	BSRF 3W1	BSRF 4W1A
X-ray source	Wiggler	Superconducting wiggler	Wiggler
Maximum magnetic intensity	1.9 T	2.6 T	1.8 T
Accelerating energy	3.5 GeV	2.5 GeV	2.5 GeV
Period length	14 cm	17 cm	139 cm
Number of periods	8	8	1
Critical energy	15.47 keV	10.81 keV	7.5 keV
Source size in horizontal	960 µm	3130 µm	1230 µm
Source size in vertical	54 µm	104 µm	420 µm

**Table 2:** Relevant parameters of the Xradia test pattern (X500-200-16)

Smallest feature size	Horizontal and vertical pitch	Precision grid pitch	Membrane thickness	Structure height	Structure material
0.5 µm	1 µm–16 µm	5 µm	1000 nm	1600 nm ± 10%	Gold

**Table 3:** Calculation results of the factors affecting the resolution in SRX imaging experiments at the BSRF 3W1 beamline

Factor affecting resolution	Calculation result
Width of the first Fresnel zone (*R*_Fre_)	1.32 µm
Resolution determined by the source PSF (*R*_src_)	0.85 µm (horizontal) × 0.16 µm (vertical)
Diffraction limit (*R*_obj_)	2.4 µm
Detector depth of field (DOF)	49 µm → 50 µm (thickness of the scintillator)
Camera resolution of projections (*R*_cam_)	5.88 µm

**Table 4:** Calculation results of the factors affecting resolution in SRX imaging experiments at BSRF 4W1A beamline

Factor affecting resolution	Calculation result
Width of the first Fresnel zone (*R*_Fre_)	2.55 µm
Resolution determined by the source PSF (*R*_src_)	3.54 µm (horizontal) × 1.2 µm (vertical)
Diffraction limit (*R*_obj_)	6.1 µm
Detector depth of field (DOF)	247 µm → 50 µm (thickness of the scintillator)
Camera resolution of projections (*R*_cam_)	7.14 µm

**Table 5:** Calculation results of the translational error in visible-light imaging experiments SD, *e*/*M*′, *R*_obj_ and *R*_cam_ represent the sample-to-detector distance, effective pixel size, diffraction limit and camera resolution, respectively. T_H and T_V represent the maximum translational error in the horizontal and vertical, respectively. The unit ‘pixel’ refers to the pixel size of the projections for each group of experiments.

	Objective									
Camera	*M*′	NA	*e*/*M*′ (µm)	SD (mm)	*R*_obj_ (µm)	*R*_cam_ (µm)	Mode	Step size (µm)	T_H (pixel)	T_V (pixel)
Andor	2×	0.08	6.5	6.2	4.2	13	2 × 2	3.25	0.01	0.02
Andor	4×	0.16	3.25	13	2.1	6.5	2 × 2	1.625	0.05	0.07
Andor	2×	0.08	6.5	6.2	4.2	13	3 × 3	2.17	0.04	0.08
